# AGE DIFFERENCES IN POLITICAL ENGAGEMENT: ROLE OF SELF-RELEVANCE

**DOI:** 10.1093/geroni/igz038.2889

**Published:** 2019-11-08

**Authors:** Tze Kiu Wong, Helene H Fung

**Affiliations:** 1 The Chinese University of Hong Kong, Hong Kong, Hong Kong; 2 Department of Psychology, The Chinese University of Hong Kong, Hong Kong, Not Applicable, Hong Kong

## Abstract

Previous studies usually found that older people are less politically engaged than younger adults, especially when considering political behavior other than voting. The current study extends the Selective Engagement hypothesis (Hess, 2014) to political engagement. 81 younger adults and 79 older adults rated 8 issues on self-relevance and their willingness to engage in political discussion, arguments and collective action on each issue. The predicted moderating effect of self-relevance was not found, but older people indeed are more willing to discuss (B = 0.07, p = 0.027) and argue with others on more self-relevant issues (B = 0.06, p = 0.031). Perceived cost of collective action was found to be a moderator, such that self-relevance was less important than other factors for high-cost actions (B = -0.016, p = 0.013). The current research sheds light on potential ways to increase older adults’ engagement in social issues.

